# Underdiagnosis of Hereditary Colorectal Cancers Among Medicare Patients: Genetic Testing Criteria for Lynch Syndrome Miss the Mark

**DOI:** 10.1200/PO.21.00132

**Published:** 2021-07-01

**Authors:** Charles Muller, Sarah M. Nielsen, Kathryn E. Hatchell, Shan Yang, Scott T. Michalski, Barbara Hamlington, Robert L. Nussbaum, Edward D. Esplin, Sonia S. Kupfer

**Affiliations:** ^1^ Section of Gastroenterology, Hepatology, and Nutrition, Department of Medicine, University of Chicago, Chicago, IL; ^2^ Invitae Corporation, San Francisco, CA

## Abstract

**PURPOSE:**

Strict clinical criteria used by Medicare for germline testing for Lynch syndrome (LS) could lead to missed diagnoses of hereditary cancer syndromes given variable individual and family phenotypes. The aim of this study was to compare rates and spectrum of pathogenic or likely pathogenic (P/LP) variants in LS and other hereditary cancer genes on the basis of meeting Medicare LS testing criteria.

**METHODS:**

Retrospective review of Medicare beneficiaries who had multigene panel testing with an indication of personal or family history of colorectal cancer (CRC) was performed. Ordering providers determined if Medicare LS criteria were met. The results of genetic testing were compared on the basis of whether or not Medicare testing criteria were met.

**RESULTS:**

Among 639 Medicare beneficiaries, 495 (77.5%) met testing criteria. Overall rates of P/LP variant identification were similar between those meeting and not meeting testing criteria (18.4% *v* 11.8%; *P* = .06). LS was diagnosed more frequently among those meeting testing criteria (10.1% *v* 4.9%; *P* = .05). No statistical differences were found in rates of P/LP variant identification for non-LS CRC genes (5.3% *v* 5.6%; *P* = .89) or non-CRC genes (4.2% *v* 2.1%; *P* = .23). *PMS2*, *MUTYH*, and *ATM* P/LP variants were found at higher rates among those outside of criteria.

**CONCLUSION:**

Among Medicare beneficiaries undergoing genetic testing for suspected LS, rates of P/LP variants in actionable cancer genes were similar regardless of whether testing criteria were met. Current testing criteria fail to identify individuals with P/LP variants in *PMS2* and other actionable cancer genes. Relaxing LS testing criteria could improve identification of individuals with hereditary cancer syndromes among Medicare beneficiaries.

## INTRODUCTION

Lynch syndrome (LS) is one of the most common hereditary cancer syndromes with an estimated prevalence of one in 279.^1^ LS is due to germline pathogenic or likely pathogenic (P/LP) variants in *MLH1*, *MSH2*, *MSH6*, *PMS2*, or *EPCAM*^2^ that lead to increased lifetime risks of colorectal cancer (CRC) and endometrial cancer as well as gastric, ovarian, pancreas, small bowel, biliary tract, ureter and renal pelvis, and brain cancers, sebaceous gland adenomas, and keratoacanthomas.^3^ LS-associated cancers have characteristic features of microsatellite instability or loss of mismatch repair (MMR) protein expression that can be assessed by tumor testing.^4,5^ Identification of individuals and families with LS enables intensive surveillance for early cancer detection, prevention, and targeted therapeutics.

**CONTEXT**

**Key Objective**
In the era of next-generation sequencing with widely available multigene panel testing for hereditary cancers, strict clinical criteria could pose a barrier to identification of individuals with actionable hereditary cancer variants. This retrospective analysis of germline testing for suspected Lynch syndrome (LS) in Medicare patients sought to determine differences in yield of testing among those meeting and not meeting Medicare criteria for LS testing.
**Knowledge Generated**
Among Medicare patients undergoing germline testing for suspected LS, no significant differences were seen in rates of overall pathogenic or likely pathogenic variants among those meeting and not meeting Medicare criteria for testing. Actionable variants in *PMS2*, *MUTYH*, and *ATM* were found more frequently among individuals not meeting criteria.
**Relevance**
Clinical criteria used by Medicare are insensitive for identification of many individuals at risk for LS and other hereditary cancer syndromes. Relaxing criteria for testing may help improve identification of individuals with hereditary cancers.

Traditionally, hereditary cancer risk assessment has involved performing germline genetic testing in selected patients who meet strict phenotypic or family history criteria,^5-7^ which has been shown to lead to underdiagnosis of individuals with hereditary cancer syndromes.^8,9^ The advent of multigene panel testing (MGPT) using next-generation sequencing (NGS) has enabled testing of multiple cancer susceptibility genes in parallel and has highlighted limitations of traditional genetic testing criteria for cancer risk assessment. Previous studies of MGPT in individuals at risk for LS or unselected patients with CRC found actionable P/LP variants in genes not associated with LS, such as *BRCA1*/*2*, among others.^10,11^

Medicare, which provides health care coverage for 62.7 million Americans,^12^ uses a set of clinical criteria to determine coverage for germline testing for LS. These criteria require a Medicare beneficiary to be affected by an LS-associated cancer, have abnormal tumor testing (or confirm that tissue is not available), and meet specific family history and/or age criteria (Table 1).^13^ Other private insurers have adopted Medicare criteria for their own policies on genetic testing coverage for LS. However, adherence to strict Medicare testing criteria for identification of hereditary cancer syndrome can lead to underdiagnosis and missed diagnoses as recently reported for hereditary breast and ovarian cancer (HBOC) syndrome.^14^

**TABLE 1.**
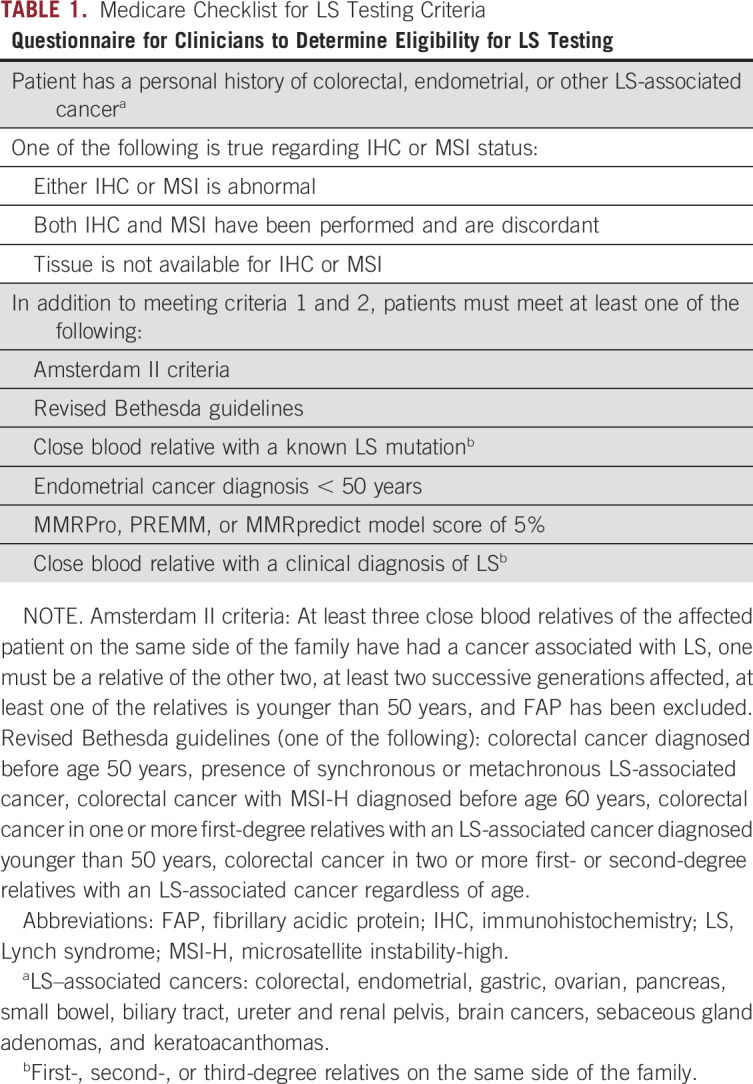
Medicare Checklist for LS Testing Criteria

The aims of this study were to compare rates and spectrum of P/LP variants in LS and other hereditary cancer genes on the basis of meeting Medicare LS testing criteria.

## METHODS

### Study Population

A retrospective review of deidentified data from a series of consecutive Medicare beneficiaries who underwent germline genetic testing with an indication of personal or family history of CRC through a single commercial laboratory (Invitae, San Francisco, CA) from September 2015 through June 2017 was performed. Genetic testing panels included, at minimum, five LS genes (*MLH1*, *MSH2*, *MSH6*, *PMS2*, and *EPCAM*). Individuals with personal or family history of CRC undergoing germline testing for known familial variants were included if they had at least the five LS genes tested as well, although this constituted a minority of included patients (2.7%). Patients who underwent broader MGPT, at the discretion of their clinician, were also included. These panels included between 20 and 83 additional genes. The price of ordering a genetic test was the same regardless of the number of genes in the panel, making test selection dependent only on patient and provider preferences. CRC genes, excluding LS genes, in these panels included *APC*, *BMPR1A*, *BLM*, *CHEK2*, *MUTYH*, *PTEN*, *SMAD4*, *STK11*, and *TP53*.

### Determination of LS Testing Criteria

During the study period (2015-2017), in an effort to study difference in yield of testing on the basis of patients' Medicare criteria status, clinicians ordering genetic testing were asked to fill out a brief checklist indicating whether an individual did or did not meet criteria for LS testing. The checklist not only was based primarily on Medicare criteria for determination of LS^13^ but also incorporated some of the more inclusive National Comprehensive Cancer Network (NCCN) criteria.^6^ Full details of the criteria included in the checklist are shown in Table 1. Personal history of CRC or other LS-associated cancer in addition to MMR-deficient tumor testing (or inability to perform tumor testing) was required for patients to be considered within testing criteria. Following completion of the checklist, clinicians were asked to indicate whether the patient met or did not meet testing criteria. For patients outside of testing criteria, additional detailed clinical justification for testing was not required.

### Genetic Testing and Variant Interpretation

Testing was performed by NGS as previously described,^15^ and variant interpretation was carried out on the basis of a refinement of guidelines from the American College of Medical Genetics and Genomics.^16^ Clinical reports were categorized as positive when a P/LP variant was identified. Reports were categorized as uncertain or negative when a variant of uncertain significance (VUS) or benign varient, likely benign, or no variants were identified.

### Statistical Analysis

Demographic data and information on genetic testing including panel type (LS only, CRC panel, or common or multicancer panel), genes included in the panel, and indication for testing were obtained from clinician-completed test requisition forms. Each patient was then categorized by their clinician as meeting or not meeting testing criteria for LS. Demographic data and outcomes of genetic testing were compared between patients who did and did not meet testing criteria using Pearson chi-square or Fisher's exact test for categorical variables and *t*-test or Mann-Whitney *U* test for continuous variables.

## RESULTS

In total, 639 unique Medicare beneficiaries undergoing genetic testing for LS were included in this study. Baseline characteristics on the basis of testing criteria are shown in Table 2. The study population had a mean age of 69.9 years (range 23-90 years) and was 61.5% female and 76.2% non-Hispanic White. Seventeen patients (2.7%) included had known familial variants. In total, 77.5% of patients met testing criteria, whereas 22.5% were categorized as not meeting testing criteria by their clinicians. Genetic testing results for those meeting and not meeting testing criteria are shown in Table 3. Overall, P/LP variants were found in 108 patients (16.9%). Of those who met testing criteria, 91 (18.4%) were found to have P/LP variants, whereas 17 (11.8%) patients not meeting criteria were found to have P/LP variants (*P* = .06). LS was diagnosed (via identification of P/LP variants in LS genes) in 10.1% of those who met testing criteria and 4.9% of those who did not (*P* = .05). Seven of 57 (12%) P/LP variants in LS genes were found in individuals not meeting testing criteria. Rates of P/LP variants associated with CRC risk (excluding LS genes) were similar among those who did and did not meet criteria (5.3% *v* 5.6%; *P* = .89). There was also no difference in the rate of identification of P/LP variants not associated with CRC among those meeting and not meeting criteria (4.2% *v* 2.1%; *P* = .23). Finally, overall rates of VUS identification were also similar between the two groups (37.8% *v* 34.0%; *P* = .41). Rates of isolated VUS identification in the absence of an actionable P/LP variant were also similar between groups (31.5% *v* 29.8%; *P* = .71).

**TABLE 2.**
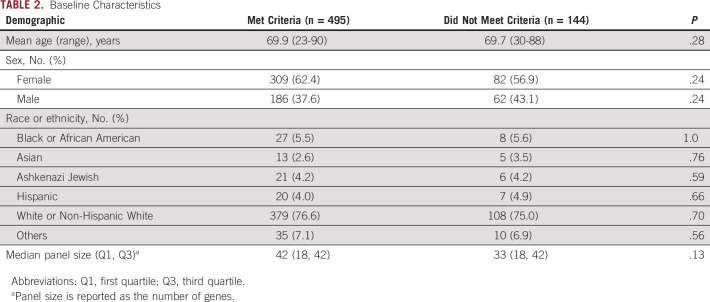
Baseline Characteristics

**TABLE 3.**
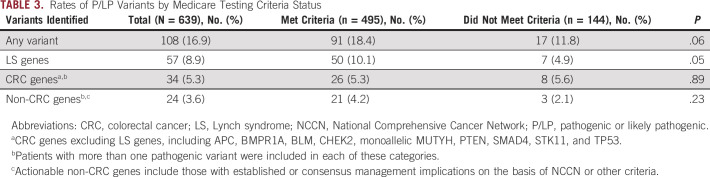
Rates of P/LP Variants by Medicare Testing Criteria Status

The type of panel ordered and rates of P/LP variants on the basis of Medicare criteria are shown in Table 4. Although testing for LS was listed as the indication for genetic testing for all included patients, a narrow panel including only the five LS genes was the least common test ordered (11.6%). A CRC panel, containing approximately 20 genes, was ordered for 25.0% of patients, whereas 63.4% had the common or multicancer gene panels ordered, containing 40-80 genes. Frequency of panel type did not differ between those who did and did not meet testing criteria (*P* = .14). More P/LP variants were found in those meeting testing criteria among those with LS gene panels ordered (35.6% *v* 13.3%), but this did not reach statistical significance (*P* = .12). For CRC and multicancer panels, rates of P/LP variant identification among those who did or did not meet criteria were similar (11.3% *v* 15.6%; *P* = .46 and 17.8% *v* 9.5%; *P* = .06, respectively).

**TABLE 4.**
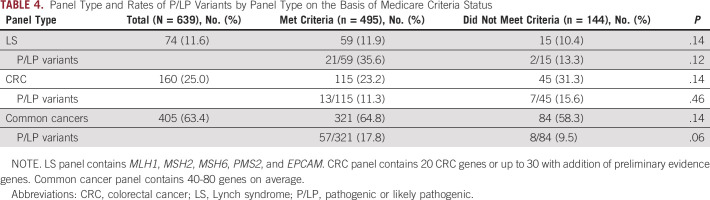
Panel Type and Rates of P/LP Variants by Panel Type on the Basis of Medicare Criteria Status

**FIG 1.**
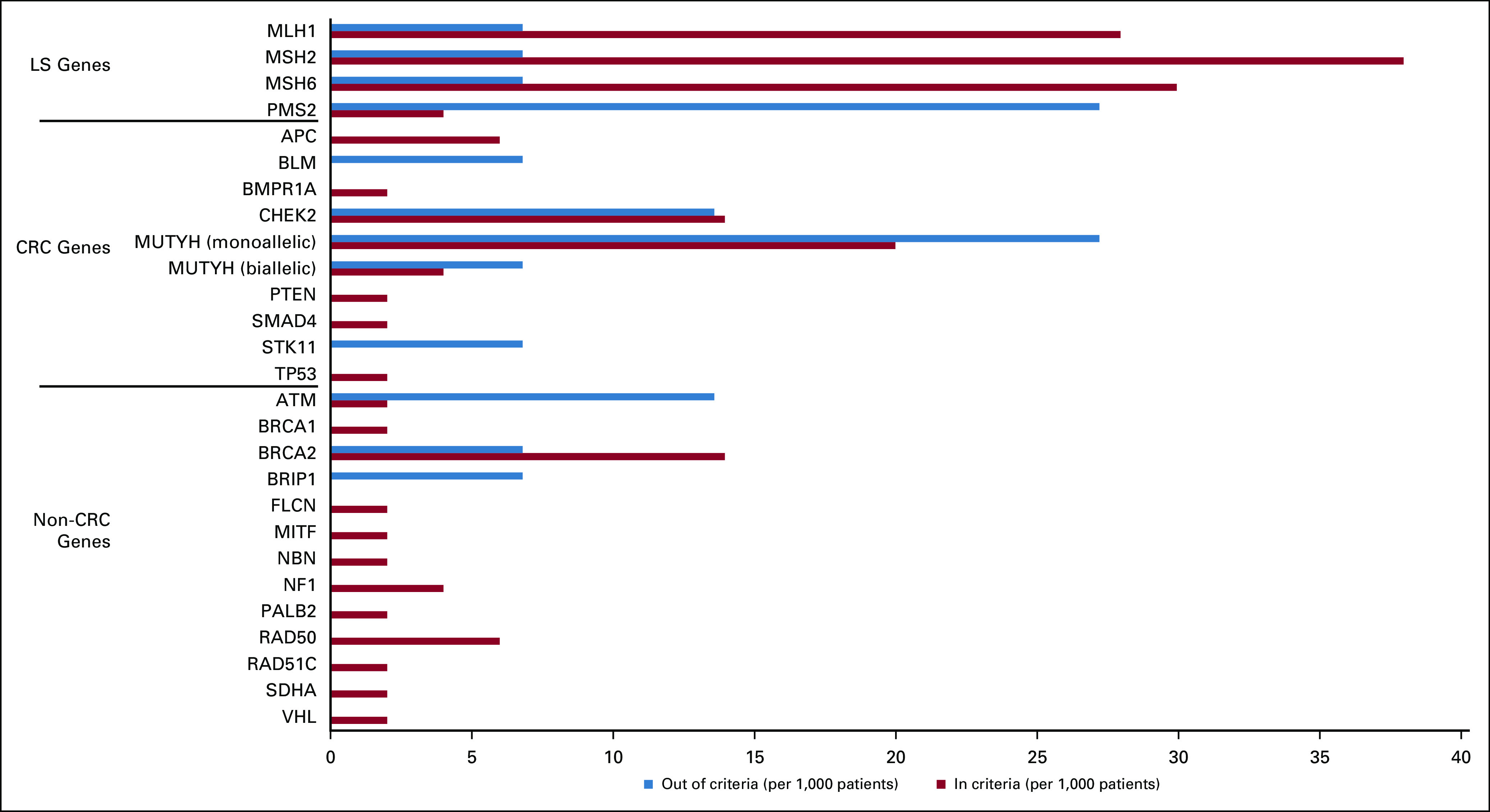
Pathogenic or likely pathogenic variants on the basis of Medicare testing criteria. Results presented as variants identified per 1,000 patients to allow for graphical comparison between two groups. CRC, colorectal cancer.

Figure 1 shows the genes in which the P/LP variants for each group of patients were identified. For both groups, P/LP variants were identified in genes that are associated with heritable CRC and in genes associated with other known cancer syndromes, such as *BRCA1* and *BRCA2* in HBOC. Almost all the genes identified with P/LP variants in this cohort had guideline-based management recommendations and are clinically actionable in the context of treatment for a patient's cancer, post-treatment surveillance, risk-reducing prophylactic measures, and screening or cascade testing for at-risk family members as shown in Appendix Table A1. A subset of 78 (12.2% of the total cohort) patients had germline findings, making them potentially eligible for precision therapy or clinical treatment trials (eg, platinum chemotherapy, poly-ADP ribose polymerase inhibitors, checkpoint inhibitors, etc), 13 of whom (16.7%) did not meet criteria. Notably, P/LP variants were found for *PMS2*, *MUTYH* (monoallelic), and *ATM* with greater frequency among individuals not meeting criteria than those meeting criteria.

## DISCUSSION

This large observational study of Medicare beneficiaries undergoing MGPT for LS demonstrated statistically similar rates of P/LP variants among patients who met Medicare testing criteria compared with those who did not meet criteria. Although LS was diagnosed more frequently among those meeting criteria, 12% of patients with LS in this study did not meet testing criteria. Moreover, no statistical differences in rates of P/LP variants in genes related to other CRC syndromes or non-CRC cancer syndromes on the basis of Medicare criteria were found. In patients who did not meet Medicare criteria, the spectrum of genes with P/LP variants was different compared with those who met testing criteria. Thus, current Medicare criteria for LS underdiagnose patients with LS and miss those with P/LP variants in other actionable cancer predisposition genes.

Existing criteria for genetic testing were developed during an era in which testing was less efficient and more expensive than current NGS technologies, and therefore, limited testing strategies that maximized specificity were favored over broader testing approaches. Although Medicare testing criteria identified more individuals with P/LP variants in LS genes (10.1% *v* 4.9%), an appreciable number of individuals outside of Medicare criteria were still found to have LS. The greater frequency of LS positivity among individuals meeting criteria is possibly driven by the requirement that individuals have evidence of MMR-deficient tumors if tumor testing was performed, resulting in a population that is much more likely to have LS than individuals with CRC without proven MMR deficiency. Among individuals who met Medicare testing criteria, the most commonly affected gene was *MSH2* followed by *MSH6* and *MLH1.* Although *MSH2* and *MLH1* are highly penetrant and expected to represent a large proportion of classic LS families, the larger number of *MSH6* variants identified in this study could be due to testing of women with personal history of endometrial cancer. Among individuals with LS who did not meet Medicare criteria, the majority was found to have P/LP variants in *PMS2.* This finding is not unexpected given that *PMS2* has a considerably lower penetrance for CRC than other LS genes^17,18^ and is less likely to result in an individual or family-level phenotype meeting LS clinical criteria.^1,19^ Moreover, *PMS2* P/LP variant prevalence has been found to be significantly higher in population-level studies than previous estimates derived from high-risk cohorts.^1^

Overall, 9% of individuals were found to have P/LP variants in non-LS genes; this did not differ on the basis of Medicare testing criteria. The rate of P/LP variants in any CRC-related gene was similar in those who met testing criteria compared with those who did not. Among individuals who met Medicare criteria, P/LP variants were identified in polyposis genes (*BMPR1A*, *SMAD4*, *PTEN*, and *APC*), whereas, among those who did not meet criteria, a larger proportion of variants were identified in *ATM*, *STK11*, and monoallelic or biallelic *MUTYH*. Reasons for these differences could be related to higher rates of family history of CRC and endometrial cancers among individuals who met criteria compared with those who did not meet criteria, although this could not be evaluated on the basis of lack of granular family history in this data set. The fact that biallelic *MUTYH* mutations were found more commonly among individuals not meeting Medicare criteria may be due to less family history of cancer among autosomal recessive conditions. Medicare criteria, which rely heavily on family history data, thus potentially fail to identify autosomal recessive CRC genes such as *MUTYH*, *NTHL1*, *MSH3*, and *MLH3*.

Although rates of P/LP variants in non-CRC cancer predisposition genes were numerically higher among those meeting criteria than those not meeting criteria (4.2% *v* 2.1%), this did not reach statistical significance. MGPT has yielded unexpected pathogenic variants that challenge conventional testing criteria and understanding of genotype-phenotype associations. This principle has been exemplified in studies showing overlapping phenotypes of the most common hereditary syndromes, LS, and HBOC.^10,19-21^ One study showed that 1.2% of individuals with suspected LS carried *BRCA1*/2 P/LP variants (of whom only one third met NCCN HBOC criteria),^10^ whereas another study found that 22% of individuals with LS met NCCN HBOC testing criteria.^19^ In the present study, of the 108 patients with P/LP variants, nine (8.2%) were carriers of variants in *BRCA1* or *BRCA2,* of whom Medicare testing criteria identified all *BRCA1* carriers but only a subset of *BRCA2* carriers. These results underscore atypical phenotypes among carriers of *BRCA1*/*2* that could appear similar to those of LS.

A theoretical pitfall of the shotgun approach of widespread use of MGPT performed outside strict clinical criteria is the increasing frequency of VUS identification with resultant uncertainty on behalf of clinicians regarding interpretation or management and added anxiety for patients and family members. Our study, however, showed similar rates of VUS identification regardless of whether the patient met criteria or not. Although previous studies have shown that VUS identification is a function of increasing panel size with more genes tested,^22,23^ the benefit of increased identification of actionable variants likely offsets any potential untoward effects of VUS identification.

This observational study allowed for characterization of genetic testing patterns and outcomes using liberalized criteria for testing in real-world practice. Indeed, most patients were tested using an MGPT rather than a disease-focused panel. The determination of whether a patient met Medicare's LS testing criteria for this study was made by individual ordering clinicians. The inability to verify whether patients in this study did or did not meet criteria for testing, or what they lacked to meet criteria, is a limitation. However, the provision of a straightforward checklist outlining criteria likely minimized inaccurate criteria determination by clinicians.

Additionally, in an effort to remove barriers to genetic testing, a requirement for detailed personal or family history when ordering was not required of providers. The variable and limited clinical information about the patients who did not meet testing criteria resulted in inability to ascertain rationale for LS testing in these patients, which is an additional limitation of the observational study design. Although all patients included had either a personal or family history of CRC, the proportion of patients who were personally affected by CRC versus those with a family history was not able to be ascertained. Medicare criteria, which require that a patient has a personal history of cancer in addition to a suggestive family history and supportive tumor features before testing, are stricter, for example, than those set by the NCCN (and modified annually).^6^ Cancer patients with a less penetrant family-level phenotype or unaffected individuals with a strong family history of cancer could face barriers to testing with this algorithm. Given that all the individuals in this study were recommended to undergo genetic testing by their providers, the cohort of patients not meeting criteria satisfied clinical suspicion for LS or other hereditary cancer syndromes and thus may be more likely to harbor pathogenic variants in hereditary cancer genes than the Medicare population at large. Although this group is therefore likely at higher risk than the average patient with CRC, the 11.8% rate of P/LP variant identification is not markedly higher than 10% rate seen in previous studies of unselected patients with CRC.^11^ Although little is known about the rate of P/LP variants in unselected individuals in the Medicare population specifically, future studies with granular data on personal and family history and a clearer explanation of indication for testing could better define which Medicare criteria most impede identification of those at risk for hereditary cancers and help guide testing strategies.

In conclusion, this large study of genetic testing in Medicare beneficiaries demonstrated that existing criteria for LS fail to detect actionable P/LP variants in LS and other hereditary cancer syndromes in a number of individuals. The current study suggests that germline testing of all patients with a personal history of CRC, as implemented by the INTERCEPT study,^15^ would substantially increase the discovery of clinically actionable findings in Medicare patients. In fact, the results of our study suggest that such a strategy would increase by up to 64% the number of patients identified to have a clinically actionable genetic test result. These results suggest that relaxing the genetic testing criteria in the Medicare population to include all patients with a personal history of CRC or other LS-related cancer (consistent with the Medical Policy of the largest commercial insurer in the United States)^24^ would improve diagnosis of hereditary cancer syndromes, and patient opportunities for germline-based precision therapy, without affecting rates of negative testing or identification of VUS.

## Figures and Tables

**TABLE A1.**
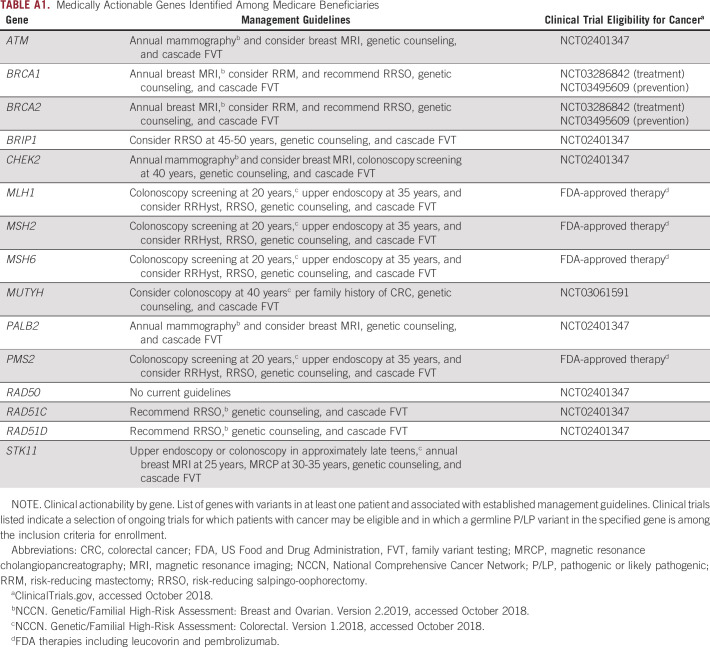
Medically Actionable Genes Identified Among Medicare Beneficiaries
